# Potential role of miR-9 and miR-223 in recurrent ovarian cancer

**DOI:** 10.1186/1476-4598-7-35

**Published:** 2008-04-28

**Authors:** Alexandros Laios, Sharon O'Toole, Richard Flavin, Cara Martin, Lynn Kelly, Martina Ring, Stephen P Finn, Ciara Barrett, Massimo Loda, Noreen Gleeson, Tom D'Arcy, Eamonn McGuinness, Orla Sheils, Brian Sheppard, John O' Leary

**Affiliations:** 1 Department of Obstetrics and Gynaecology, Trinity College Dublin, Trinity Centre for Health Sciences, St. James's Hospital, Dublin 8, Ireland; 2 Department of Histopathology, Trinity College Dublin, Trinity Centre for Health Sciences, St James's Hospital, Dublin 8, Ireland; 3 The Dana Faber Cancer Institute, Harvard Medical School, Boston, MA, USA

## Abstract

**Background:**

MicroRNAs (miRNAs) are small, noncoding RNAs that negatively regulate gene expression by binding to target mRNAs. miRNAs have not been comprehensively studied in recurrent ovarian cancer, yet an incurable disease.

**Results:**

Using real-time RT-PCR, we obtained distinct miRNA expression profiles between primary and recurrent serous papillary ovarian adenocarcinomas (n = 6) in a subset of samples previously used in a transcriptome approach. Expression levels of top dysregulated miRNA genes, miR-223 and miR-9, were examined using TaqMan PCR in independent cohorts of fresh frozen (n = 18) and FFPE serous ovarian tumours (n = 22). Concordance was observed on TaqMan analysis for miR-223 and miR-9 between the training cohort and the independent test cohorts. Target prediction analysis for the above miRNA "recurrent metastatic signature" identified genes previously validated in our transcriptome study. Common biological pathways well characterised in ovarian cancer were shared by miR-9 and miR-223 lists of predicted target genes. We provide strong evidence that miR-9 acts as a putative tumour suppressor gene in recurrent ovarian cancer. Components of the miRNA processing machinery, such as Dicer and Drosha are not responsible for miRNA deregulation in recurrent ovarian cancer, as deluded by TaqMan and immunohistochemistry.

**Conclusion:**

We propose a miRNA model for the molecular pathogenesis of recurrent ovarian cancer. Some of the differentially deregulated miRNAs identified correlate with our previous transcriptome findings. Based on integrated transcriptome and miRNA analysis, miR-9 and miR-223 can be of potential importance as biomarkers in recurrent ovarian cancer.

## Introduction

Ovarian cancer is the leading cause of death from gynaecological malignancy in the western world [1]. Ovarian serous adenocarcinomas (OSC) are the commonest histotype and account for almost 50% of malignant neoplasms [2]. The majority of cases present in advanced stages and are treated with surgery and systemic chemotherapy. Current treatment is frequently followed by recurrence, which is often resistant to chemotherapy, as demonstrated by 15% long-term survivors [3]. Although focusing on known genes has already yielded new information, previously unknown noncoding RNAs, such as microRNAs (miRNAs), may also lend insight into the biology of ovarian cancer.

This new and surprisingly abundant class of RNA regulatory genes has been found to confer a novel layer of genetic regulation in cells. Active, mature miRNAs function as endogenous, highly conserved, small RNA's, 22 nucleotides long that silence gene expression by binding to target mRNAs. Their 5' end binds to its target complementary sequence in the 3'-untranslated region (3'UTR) of mRNA and given the degree of complementarity, miRNA binding appears to result in translational repression, or in some cases, cleavage of cognate mRNAs, causing partial or full silencing of the respective protein-coding genes [4]. An accumulating body of evidence reveals critical functions for miRNAs in various biological processes as diverse as proliferation, apoptosis, and cell differentiation [5] and given their diversity and abundance, miRNAs appear to functionally interact with various components of many cellular networks. Almost 30% of the human genome is estimated to be regulated by miRNAs [6]. Therefore, they should be considered one of the largest classes of gene regulators.

Functional miRNAs are excised from long endogenous transcripts by the sequential action of a pair of endonucleases (Drosha and Dicer) that reside in different compartments of the cell. In the nucleus, the primary microRNA (pri-miRNA) transcript is first cleaved by Drosha, liberating an approximately 60-to-80-nucleotide-long hairpin-shaped precursor miRNA (pre-miRNA). This pre-miRNA is then exported from the nucleus to the cytoplasm, where it undergoes a further processing by the Dicer enzyme and the resulting duplex is then loaded onto the RNA-induced silencing complex (RISC) in order to become more effective. Perfect base pairing between the RISC-bound miRNA and the target mRNA results in cleavage and degradation of the latter, whereas imperfect complementarity generally leads to translational repression of the target [7]. RISC recruits a multiprotein complex containing the anti-association factor eIF6. Depletion of eiF6 in human cells abrogates miRNA-mediated regulation of target protein and mRNA levels [8].

Not surprisingly, a variety of studies have linked aberrant microRNA expression to carcinogenesis where they act as both oncogenes and tumour suppressor genes [9]. Unique miRNA expression profiles have been able to classify various cancers. In one study, for example, the expression pattern of 217 microRNAs identified cancer type more accurately than messenger RNA [10].

**Figure 1:**
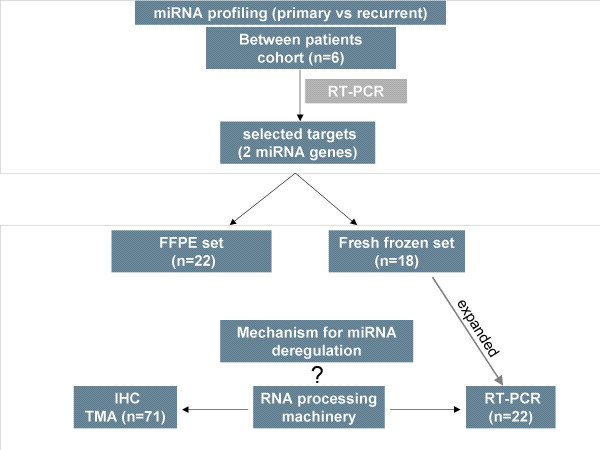
Flow chart of our study design. miRNA profiling was performed using TaqMan PCR in a homogenous set of 3 primary advanced and 3 recurrent serous papillary ovarian adenocarcinomas from different patients. Selected miRNA targets (n = 2) were examined in two independent sets of primary and recurrent fresh frozen (n = 18) and FFPE serous papillary adenocarcinomas (n = 22). We also attempted to determine whether components of the miRNA processing machinery (Dicer and Drosha) are responsible for miRNA dysregulation in recurrent ovarian cancer.

A recent study reported aberrant miRNA expression in ovarian cancers compared to normal ovary [11]. In addition, direct evidence that miRNA is of critical importance in chemoresistance of human ovarian cancer has just been published [12]. In an attempt to understand the biology of recurrent ovarian cancer, we examined the expression of 180 miRNAs in primary and recurrent serous papillary adenocarcinomas, which our group has previously interrogated in a transcriptome study in recurrence [13,14]. The aim of this study was to investigate the expression levels of miRNAs in recurrent ovarian cancer and to examine the expression levels of key components of the miRNA processing machinery. A flow chart of our experimental design is shown in Figure 1.

## Materials and methods

All samples were obtained at initial cytoreductive procedure from patients treated for ovarian cancer at St James's Hospital, Dublin, Ireland. The study had approval of the hospital ethics committee and informed consent was obtained from each patient by the research team prior to surgery. All tumors were staged according to the International Federation of Gynaecology and Obstetrics standards (FIGO).

### Patients and tissue samples

A pilot study was undertaken to examine the expression of 180 miRNAs with the objective of generating a training set.

#### Fresh frozen samples

The initial cohort (training set) consisted of 3 fresh frozen primary serous papillary (stage III and grade III) and 3 fresh frozen recurrent serous papillary ovarian adenocarcinomas of the same grade but from different optimally debulked (residual disease <0.5 cm) patients. The mean age in years for patients in the primary and the recurrent group was 63.3 (range 48–84) and 53.3 (range 45–68). The recurrent group consisted of patients for which primary surgery was performed prior to the commencement of the study. Patients had received no neoadjuvant treatment before surgery and they were mostly treated postoperatively with platinum and/or paclitaxel.

A subset of miRNAs selected from the training set was further analyzed in fresh frozen and formalin fixed paraffin embedded (FFPE) cohorts.

The additional cohort of fresh frozen tissues, which were analysed with the putative signature miRNA panel comprised 18 serous papillary adenocarcinomas, 11 primary and 7 recurrent cases.

This cohort was further expanded to 22 serous papillary adenocarcinomas -14 primary and 8 recurrent- and used to examine the relative expression levels of Dicer and Drosha. An additional 2 primary and 5 recurrent ovarian cancer patients of different histology (3 patients with mixed mullerian carcinomas, 1 patient with adenosquamous differentiation, 2 patients with poorly differentiated clear cell carcinoma and 1 patient with dedifferentiated germ cell carcinoma) were subsequently added to the cohort (n = 29).

Specimens were snap frozen on collection within 1 hour of surgery at -80°C. After tissue processing in a cryostat at -20°C, frozen sections were cut and mounted on slides. The slides were stained with H&E and examined by a pathologist to ensure >70% presence of tumour cells.

#### miRNA extraction from fresh tissues

miRNA was extracted from fresh tissues using the Ambion mirVana™ miRNA isolation kit (Ambion, Austin, TX) according to the manufacturer's instructions. Samples were placed in liquid nitrogen, ground thoroughly with a mortar and pestle and homogenised in lysis buffer. In brief, homogenised samples were lysed, followed by an acid-phenol:chloroform extraction. Total RNA samples were collected and small RNAs were purified by precipitation with ethanol. Small RNA species were immobilized on glass-fiber filters, followed by several washes and elution of small RNA with nuclease-free water.

#### Formalin-fixed paraffin-embedded (FFPE) tissues

An independent set of 22 OSC – 15 primary and 7 recurrent, classified according to the FIGO system: stage (II–III) and grade 2–3, were selected from archival FFPE tissue, between the years 1996–2006 from St James's Hospital, Dublin. H&E slides of all tumors were reviewed by a histopathologist (RF) to confirm original diagnoses.

An additional paraffin tissue microarray (TMA) of 56 primary epithelial ovarian carcinomas (EOC) and 15 recurrent ovarian carcinomas (OC) was constructed, composed of tumors arrayed in quadruplicates. Samples were age-matched to 40 normal ovaries containing normal ovarian surface epithelium (NOSE). FFPE blocks were selected that contained over 90% tumour. Slides of archival material were reviewed to determine suitable areas for the TMA construction. 4 representative areas were selected and verified by a second pathologist (CB) for core biopsy.

#### miRNA extraction from formalin-fixed paraffin-embedded (FFPE) tissues

This was performed on the above mentioned independent set of OSC (n = 22) using the Recoverall™ Total Nucleic Acid Isolation Kit (Ambion, Austin, TX). Where tumour cell density was >90% (n = 16), whole sections were taken for miRNA analysis. In cases that tumour stroma component was significantly present (n = 6), Laser Capture Microdissection (LCM) was performed to produce pure cell population for analysis. Up to 3 extractions were performed in parallel with one pellet in each extraction. Following dissection from the blocks, these preparations were deparaffinized, followed by protease digestion for 3 hours at 50°C for RNA isolation, on column DNA digestion and elution as described in the protocol.

#### Laser capture microdissection (LCM)

LCM was employed in six of the recurrent FFPE tumour samples to yield sufficient homogenous populations of malignant epithelial cells. 8 μm sections were cut from each block, dewaxed and stained with haemotoxylin and eosin (H&E). Malignant epithelial cells from the tumour papillae in each case were laser capture microdissected, using the PixCell 11™ System (Arcturus Engineering Inc., CA, USA) for subsequent expression analysis. Cells were captured according to a standard protocol as follows: Laser spot size = 30 μm, pulse power = 40 mW, pulse width = 1.5 ms, threshold voltage = 285 mV. The total number of pulses in each case was approximately 700. This yielded a tissue volume in the range of 10^-7 ^to 10^-6 ^um^3^(representing 5000 cells). Following microdissection the Capsures™ were placed in sterile Eppendorf tubes.

#### miRNA expression analysis and Real-time TaqMan PCR using full miRNA panel

miRNA expression levels were examined using the Applied Biosystems TaqMan^® ^MicroRNA Assay Human Panel Early Access kit (Applied Biosystems, Foster City, CA) consisting of 180 well characterized miRNAs. The kit uses gene-specific stem-loop reverse transcription primers and TaqMan probes to detect mature miRNA transcripts in a 2-step qRT-PCR assay. Briefly, single stranded cDNA was generated from total RNA sample by reverse transcription using the Applied Biosystems High-Capacity cDNA Archive Kit (Applied Biosystems, CA, USA) following manufacturer's protocol. RT reactions contained 10 ng of total RNA, 50 nM stem-looped RT primer, 1 × RT buffer, 0.25 mM each of dNTPs, 3.33 U/μl Multiscribe reverse transcriptase and 0.25 U/μl RNase Inhibitor. PCR amplification was carried out using sequence specific primers on the Applied Biosystems 7900 Real-Time PCR system. The reactions were incubated in a 96-well optical plate at 95°C for 10 min, following by 40 cycles of 95°C for 15 s and 60°C for 10 min. The assay was run in triplicate for each case to allow for assessment of technical variability. let-7a was used as endogenous control. Four negative controls were also used, ath-mir159a, cel-lin-4, cel-miR-2 and cel-miR-124 as they show no expression in human tissue.

#### Selected miRNA expression analysis in independent cohorts using TaqMan MicroRNA Assays

The Applied Biosystems stemloop RT/PCR kit (AB) and TaqMan microRNA Assays were used to detect and quantify selected mature miRNAs in both fresh frozen and FFPE samples, as described for the Early Access Kit protocol.

#### TaqMan gene expression analysis of Dicer and Drosha

The expanded cohort of fresh frozen samples was used for this analysis. Dicer (Assay ID: Hs00203008_m1) and Drosha (Assay ID: Hs00229023_m1) were analysed using qRT-PCR. Briefly, RNA was initially reverse transcribed using a High Capacity cDNA Archive Kit (Applied Biosystems, CA, USA) and then was amplified in a 10 μL PCR reaction according to the manufacturer's recommended protocol and amplification steps: denaturation at 95°C, followed by 40 cycles of denaturation at 95°C for 15 sec and then annealing at 60°C for 1 min. All reactions were carried out on the ABI Prism 7000 Sequence detection system (Applied Biosystems, Applera UK, Cheshire, UK) using the TaqMan^® ^Universal PCR master Mix and Assays on demand (Applied Biosystems). Relative quantitation was carried out using the ΔΔCt method for recurrent versus primary with 18S ribosomal RNA as an endogenous control. Transcript quantification was performed in triplicate for each sample.

#### Immunohistochemistry of eIF6 and Dicer on tissue microarrays (TMA)

4 μm sections of the OSC tissue array were cut and mounted on glass slides. For antigen unmasking, deparaffinized sections were boiled (eIF6) or microwaved (Dicer) in citrate buffer [10 mmol/L sodium citrate buffer (pH 6.0)] before incubation with primary antibodies. eIF6 (BD Biosciences) and Dicer (Abcam) protein levels were examined using mouse monoclonal antibodies at dilutions of 1:200 and 1:50 respectively. Antibody staining was done using the Biogenex Super Sensitive™ Link-Label IHC Detection system.

Intensity of eIF6 and Dicer immunoreactivity was recorded in the neoplastic glands for every specimen. The intensity of staining was graded on a scale from 0 to 3. "0" reflected a lack of immunoreactivity, "1" reflected weak immunoreactivity, "2" reflected moderate immunoreactivity and "3" reflected strong homogenous nucleocytoplasmic (eIF6) or cytoplasmic (Dicer) staining. Scores from all cores were averaged. Two pathologists (RF, CB) scored the stained slides.

#### Data analysis and bio-informatics in the pilot study

Relative quantitation using the ΔΔct method in recurrent versus primary was carried out [15] and fold changes were calculated for each gene. Replicates with a ct > 40 were excluded. Spotfire^® ^software [16] was used to visualize hierarchical clustering between differentially expressed genes. Unsupervised clustering was applied to the data set using the Unweighted Pair Group Method with Arithmetic Mean (UPGMA) based on Euclidean distance as the similarity measure. Target prediction analysis was carried out using the miRGen webserver. Functional classification of the data and gene ontology of the predicted mRNA targets was defined by using the PANTHER (Protein Analysis THrough Evolutionary Relationships) Classification System. A binomial statistical tool was employed to compare gene lists to a reference list (i.e. the complete human genome) to determine over- or under-representation of PANTHER classification categories [17].

Correlations of miRNA expression levels were carried out using student's t-test for age and CA125 and Fischer's test for debulking status and chemosensitivity with significance level set at p < 0.05. Chemosensitivity was defined according to the WHO criteria as complete remission after 6 months post completion of chemotherapy.

All statistical analysis of immunohistochemical studies was performed using the Analyse-It™ Software Ltd. Two sample comparisons (malignant versus normal) were performed with the Mann-Whitney rank sum test.

## Results

### A miRNA expression signature discriminates primary and recurrent ovarian cancers (training set)

**Figure 2:**
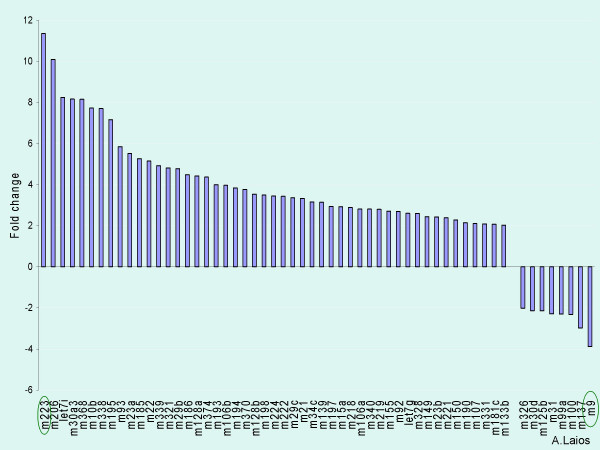
Differentially expressed miRNAs in recurrent versus primary ovarian cancers. Relative fold changes are displayed on the y axis and the miRNAs are on the x axis. The most significant altered expression was observed for mir-223 (up) and mir-9 (down).

Using the early Access kit distinct expression signatures were identified between primary and recurrent ovarian cancers. 60 miRNAs were greater than 2-fold deregulated between primary and recurrent cases (See Additional file 1). A marked upregulation of miRNAs in recurrent tumours compared to primary was observed (52 miRNAs showed overexpression and 8 underexpression of >2 fold in recurrent versus primary tumours) (Figure 2). 12 miRNAs were not detectable in the ovarian samples. mir-223 (up) and mir-9 (down) were the top dysregulated species in the recurrent versus primary samples. Notably, we observed expression of miRNAs previously reported in other human cancers such as miR-155 [18], miR-21 [19], miR-221 and miR-222 [20].

**Figure 3:**
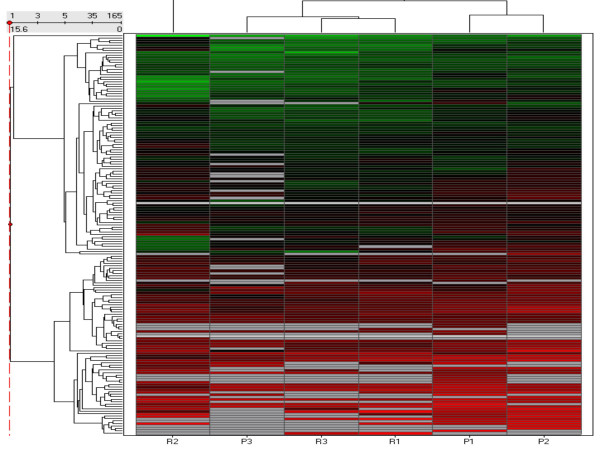
Unsupervised hierarchical cluster heatmap based on differential miRNA expression patterns identified in the initial cohort. Vertical bars represent the samples and the horizontal bars represent the miRNA genes. Green bars reflect downregulated genes and red bars upregulated genes. Interestingly, P3 clusters with the recurrent samples on the left of the heatmap. P3 relapsed within 6 months post completion of treatment and should be considered "chemoresistant" P, primary tumours; R, recurrent tumors.

However, unsupervised hierarchical clustering of the samples in the training cohort, based on differentially expressed miRNA did not clearly separate primary and recurrent tumours. Interestingly, it segregated primary "chemosensitive" tumours while the primary "chemoresistant" tumour clustered together with the recurrent ones (Figure 3).

### miRNA expression in an independent set of fresh frozen tumours of the same histology

**Figure 4:**
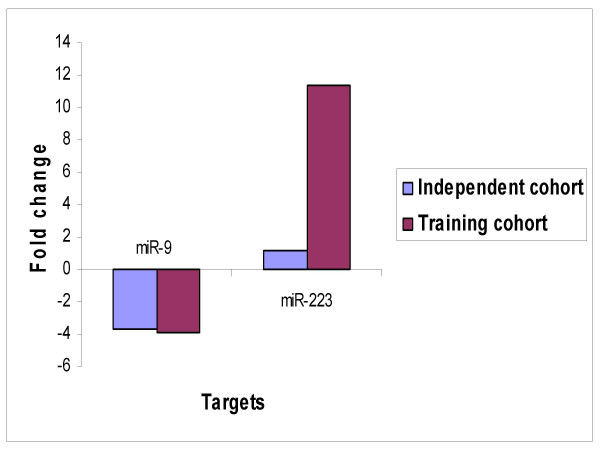
Independent TaqMan^® ^PCR validation of top dysregulated miRNAs. Concordance was observed on TaqMan analysis for miR223 and miR9 between the training and expanded independent cohort of serous ovarian carcinomas (comprised of snap frozen tissues).

To determine the potential importance of the initially identified miRNAs, real time PCR was performed in an independent set of 11 advanced primary and 7 recurrent serous papillary adenocarcinomas. Based on initial training set data, 2 miRNAs (miR-9 and miR-223) were selected from the signature panel of 60 miRNAs according to a) their degree of dysregulation (they demonstrated the greatest changes in expression levels) b) their predicted target miRNAs (Table 1). A similar pattern of dysregulation was observed for miR-9 and miR-223 on TaqMan analysis (Figure 4).

**Table 1:** List of top dysregulated mirnas and their predicted gene targets.

**MiRNA**	**Fold change**	**Expression in recuurent vs Primary**	**MiRGen**	**Transcriptome study**^13^
miR-223	11.35571231	Upregulated	FGFR2, EGF, S100A3, KRAS, TGFB2, IFNB1, SPINK5, E2F1	SEPTIN6, MMP9, USF2
miR-9	3.886456405	Downregulated	FGF18, FGF10, BCL2, BCL6, BRAF, CLDN14, CLDN6, SEPTIN10	ZNF, PVRL2, LASS4, BCL2, CLDN, FGF

These represent genes predicted by MiRGen well characterised in ovarian carcinogenesis and also genes identified in an earlier study performed by our group [13].

### miRNA expression in FFPE ovarian adenocarcinomas correlates with training cohort

**Figure 5:**
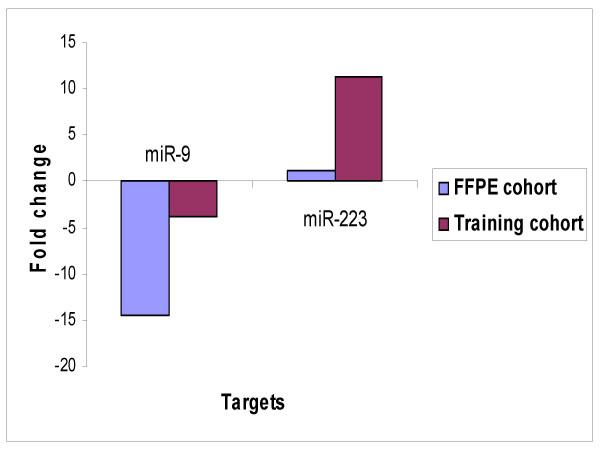
TaqMan^® ^PCR validation in training cohort and FFPE cohort. The relative quantitation from TaqMan^® ^in recurrent vs primary tumours are plotted for both cohorts.

miRNA expression analysis for the 2 selected miRNAs was carried out using real-time PCR in an independent cohort of 15 primary and 7 recurrent FFPE tissues (n = 22). TaqMan data corroborated with the results obtained from the initial cohort in so far as miR-223 was upregulated and miR-9 downregulated (Figure 5).

### Predicted and confirmed mRNA targets

To investigate the biological consequence of altered miRNAs, we analyzed the predicted gene targets of the top deregulated miRNA genes identified in our cohort. The analysis was conducted using the miRGen webserver, an online tool that takes sets of target genes, predicted by the individual programs and presents compiled lists of the intersection of miRanda and TargetScan algorithms (See Table 1).

### miR-9 and miR-223 gene prediction analysis identifies common significant biological pathways

**Figure 6:**
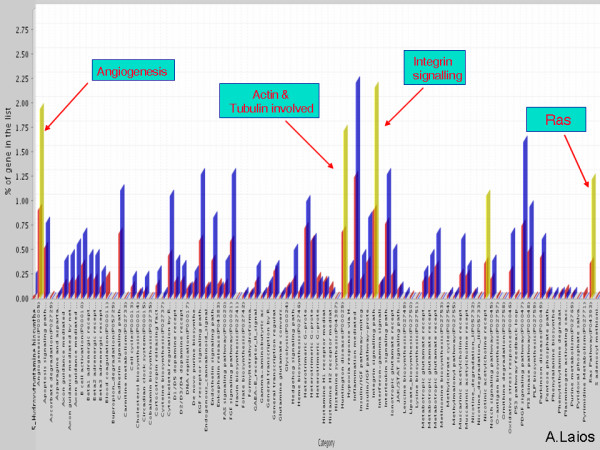
Figure illustrating common biological pathways identified when the lists of predicted targets for miR-9 and miR-223 were exported in the Panther classification system and examined against the H. sapiens reference list to determine the percentage of genes compared to what expected. Common pathways between the top dysregulated genes were significant at a p < 0.05 as highlighted by the orange bars.

The list of predicted targets for both miR-223 and miR-9 was further uploaded into Panther classification system and examined against the H. sapiens reference list to determine what percentage of genes were present compared to that expected and to determine whether any relevant biological pathways were statistically significant at a p value of 0.05. Common significant pathways were observed for the "miR-223 list" and "miR-9 list" of predicted gene targets at a p < 0.05; namely angiogenesis, Ras, integrin signalling and a pathway involving actin and tubulin (Figure 6).

Statistical analysis of clinical variables including age, CA125, debulking status and chemosensitivity did not prove significant when correlated with expression levels of miR-223 and miR-9.

### TaqMan analysis of Dicer and Drosha

In order to elucidate the mechanism of miRNA dysregulation in the recurrence of ovarian cancer we characterised the alteration in expression of genes encoding proteins of the miRNA machinery, namely Dicer and Drosha, in 14 primary and 8 recurrent fresh frozen OSC, using qRT-PCR. Fold changes less than 2 were observed for both genes in our initial cohort and our extended set of serous papillary adenocarcinomas (Relative fold changes primary vs recurrent: Drosha FC = 0.91, Dicer FC = 1.19). We further expanded our test set to 16 primary and 13 recurrent samples by adding tumours of other than OSC histological subtypes (See Materials and Methods). Fold changes of 1.78 and 1.49 were observed for Drosha and Dicer respectively, but were not significant.

### Immunohistochemical analysis of eIF6 and Dicer expression in primary versus recurrent serous ovarian adenocarcinomas

**Figure 7:**
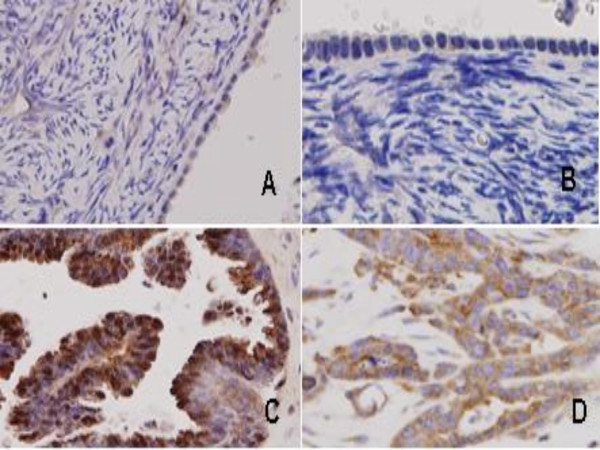
Expression of eIF6 and Dicer in normal ovarian surface epithelium and ovarian serous adenocarcinoma (×40): No staining ('0') for eIF6 (A) and Dicer (B) in normal ovarian surface epithelium. Strong ('3') staining for eIF6 (C) and Dicer (D) in ovarian serous adenocarcinomas.

**Figure 8:**
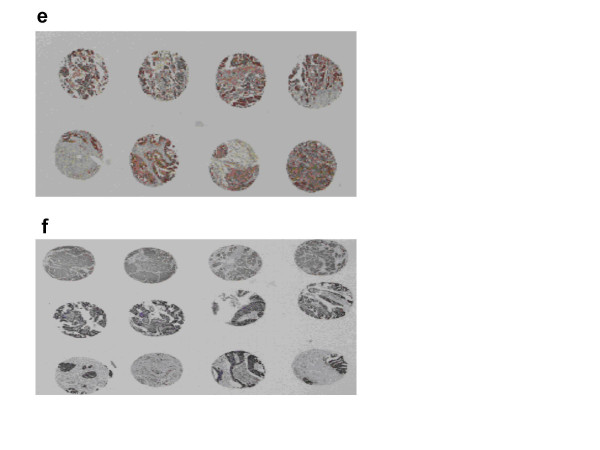
Global staining pattern for eIF6 (e) and Dicer (f) in a TMA of primary serous papillary ovarian adenocarcinomas. Global staining pattern for eiF6 and Dicer in the TMA of recurrent ovarian tumours is not shown. Analysis was based on relative expression levels of eIF6 and Dicer in primary vs recurrent tumours.

We examined the relative expression levels of eIF6 and Dicer on TMAs including 56 primary OSC and 15 recurrent ovarian tumours and determined whether any correlation exists between the two. In this respect, we wanted to examine functionality of the "mature" sector of the miRNA machinery at a protein level. No statistically significant observations were made (Figures 7 and 8).

## Discussion

This study demonstrates discrete miRNA signatures between primary and recurrent serous papillary adenocarcinomas on a histologically homogenous set of ovarian tissues from different patients. To our knowledge, this is the first ever study to examine the expression levels of miRNAs in recurrent cancer.

Real-time PCR can be employed to quantify miRNA expression profiles and study the potential function of miRNAs as well as miRNA precursors in cancer pathogenesis [21]. We utilized a novel stem-looped TaqMan RT-PCR method to quantify the expression profiles of 180 mature miRNAs in a collection of primary and recurrent serous papillary adenocarcinomas. This method offers several distinct advantages over conventional miRNA detection methods including a higher sensitivity, enhancing specificity for mature miRNAs and a fast and simple methodology [22].

We used a twofold change as a cut-off for miRNA differential expression in our training set. One might argue, much smaller changes could also be significant, reflecting the fact that miRNAs target many genes of even similar function. In this regard, disruption of cooperating genes can be a likely event with subtle miRNA expression [23]. However, unsupervised hierarchical clustering failed to segregate samples in primary and recurrent cohorts (3vs3). Instead, a single primary "chemoresistant" tumour clustered together with the recurrent ones suggesting inherent genetic differences between chemosensitive and chemoresistant groups. This finding enforces the potential advantage of utilizing miRNA expression patterns to predict relapse in ovarian cancer.

Because the expression of some miRNAs is highly tissue specific [24], it was actually expected that not all of the miRNAs would be detected in ovarian cancer samples. Furthermore, because of the tissue specificity, different sets of miRNAs are likely to be deregulated in cancers of different cellular origin, although it has been reported that the miRNA signatures of different cancer types could share some individual miRNAs [25]. Of the miRNAs we detected here as dysregulated, many are similarly dysregulated in other cancers: down-regulation of miR-125b in breast cancer [26], the let-7 miRNA family in lung cancer [27], miR-21 which activates PTEN in hepatocellular cancer [19], miR-155 in B cell lymphomas [28], miR-221 and miR-222 that clearly differentiate papillary thyroid carcinomas (PTC) from normal thyroid tissues (See Additional file 1). miR-221 and miR-222 block endothelial cell migration, proliferation and angiogenesis in vitro by targeting the stem cell factor receptor c-Kit [29]. miR-21 expression was shared by the signatures of six solid cancers (breast, colon, lung, pancreas, stomach, prostate) in a large scale miRome analysis [25] suggesting that miRNAs are involved in common molecular pathways. However, the majority of the differentially expressed miRNAs detected in this study have not been commonly reported to be deregulated in other cancers, implying that primary and recurrent ovarian cancers may have miRNA signatures specific for this cancer type.

Real time PCR was further employed to detect expression levels of a subset of miRNAs in an expanded cohort of fresh frozen serous papillary ovarian adenocarcinomas. Selected miRNAs comprised miR-223 (up) and miR-9 (down), which had demonstrated the greatest changes in expression levels in the initial cohort. Similar patterns of altered expression were observed for the top dysregulated genes (Figure 4).

We then set out to determine the expression patterns of selected miRNAs across a large panel of FFPE pathological tissues representing primary and recurrent serous ovarian adenocarcinomas, routinely used in hospital pathology laboratories. Unlike mRNAs, miRNAs remain largely intact in these routinely collected clinical samples [30]. "Recurrent" samples are rather limited, thus valuable, in common clinical practice therefore, LCM was performed in a subset of recurrent samples, as it can harvest tumour cells, in order to give histologically pure enriched cell populations [31]. Few earlier studies have addressed the expression characteristics of miRNA genes from FFPE tissues [30]. Our results suggest human archival tissue is a reliable source of study material for analyzing miRNA expression levels.

This miRNA "recurrent metastatic signature", comprised miR-223 and miR-9 may deserve some special consideration. miR-223 was the most upregulated in recurrent cancers when compared to primary. Recent work by Chen et al. identified among others miR-223 as a murine hematopoietic-specific miRNA and showed functionality of these miRNA through in vitro and in vivo overexpression studies [32]. miR-223 was highly expressed in cell lines of myeloid origin suggesting important regulatory roles in human hematopoiesis and oncogenesis [33]. More recently, it has been reported that miR-223 is a key member of a regulatory circuit that controls granulocytic differentiation and clinical response of acute promyelocytic leukemia (APL) blasts to all-trans retinoic acid (ATRA) [34]. ATRAs appear to be new promising drugs as they have been shown to arrest growth of ovarian carcinoma cells [35]. The potential role of miR-223 in immune and anti-inflammatory response has been recently described [36].

miR-9 was the most downregulated in recurrent cancers and appears to play a functional role in the determination of neural fates in embryonic stem (ES) cell differentiation [37]. miR-9 was also transcriptionally downregulated in a methylation-dependent way, in early breast cancer development [38].

miRNAs have been recently implicated in the development of ovarian cancer [11]. Specifically, in the serous histotype, 37 of 41 differentially expressed miRNAs were down-regulated relative to normal ovary. Notably, miR-9 downregulation was commonly shared between the serous and endometrioid histotypes compared to normal ovaries. The absence of other common miRNAs between the two studies, including miR-223 that was not identified in that study could be due to different experimental design (we carried out miRNA profiling in primary vs recurrent and not malignant vs normal) but might also reflect different experimental approaches or tumour heterogeneity. It also suggests that different miRNAs are involved in different stages of ovarian carcinogenesis. Iorio et al also identified miR-214 as "serous specific", which has been recently linked with chemoresistance in ovarian cancer by inducing cisplatin resistance through targeting the PTEN/Akt pathway [12]. miR-214 was also not included in our 60 miRNA-signature of dysregulated genes, possibly due to different selection criteria of control cells [39] or tumour heterogeneity.

Our team has previously examined gene expression levels in the training sample and identified over and under represented genes with full functional annotation [14]. In the current study, miRGen webserver was used to determine lists of putative mRNA targets of differentially expressed miRNAs. Although these lists may contain an unpredictable number of false positives, miRGen appears superior to other approaches, providing higher specificity for individual miRNAs [40]. It was interesting to observe that some of the differentially regulated miRNAs in this study correlate with our transcriptome findings [13] (See also Table 1) as rather few studies have attempted to link mRNA with miRNA data in different cancers [41,42]. We provide strong evidence that miR-9 is a putative tumour suppressor gene given that predicted targets for miR-9 include members of the Claudin and FGF family that were upregulated in the recurrent tumours.

Common significant biological pathways involving predicted gene targets for miR-9 and miR-223 were identified using PANTHER. It appears that these pathways contain traits that distinguish primary and recurrent ovarian cancers. They have all previously been well characterised in ovarian cancer and chemoresistance. In this respect, if downregulated miRNAs are expected to activate overexpression of mRNA targets, it is fair to suggest that miR-9 downregulation can possibly lead to drug resistance.

The target list for miR-223 and miR-9 was interestingly enriched for genes involved in FGF cell signaling (FGFR2 and FGF respectively), which is in concordance with our transcriptome results [13]. This is also consistent with the very latest discovery that FGF signaling is crucial for collective invasion of carcinoma cells following the tracks generated by fibroblasts [43].

The mechanism of miRNA deregulation in recurrent ovarian cancer has not been thoroughly exploited. Fold changes <2 were observed for Dicer and Drosha at a mRNA level in our initial cohort and extended set of tumours with same histology. Inclusion of non serous tumours in the previously homogenous set conferred no statistical significance. To determine whether the observed gene expression pattern was altered at the protein level, primary and recurrent ovarian tumors (n = 71) were analysed immunohistochemically on TMA constructs containing multiple sections, using specific antibodies for Dicer and EIF6. Both proteins are localized in the cytoplasm and have roles in the processing of mature miRNAs.

Our results show that Dicer, Drosha and eIF6 may not have a substantive role in recurrent ovarian cancer and may only be involved in early tumorigenesis as shown by Chiosea et al [44]. We are unsure whether this represents a delay in processing or a complete block on the miRNA machinery, but it is possible that altered miRNA machinery is still functional and other components also participate, such as argonautes and p-bodies [45]. Our data do not exclude other important mechanisms, such as DNA copy number alterations [46] or disrupted epigenetic regulation [7], but rather suggest a multistep model for the control of miRNA dysregulation. The exact nature of the regulatory mechanism awaits further investigation.

## Conclusion

**Figure 9:**
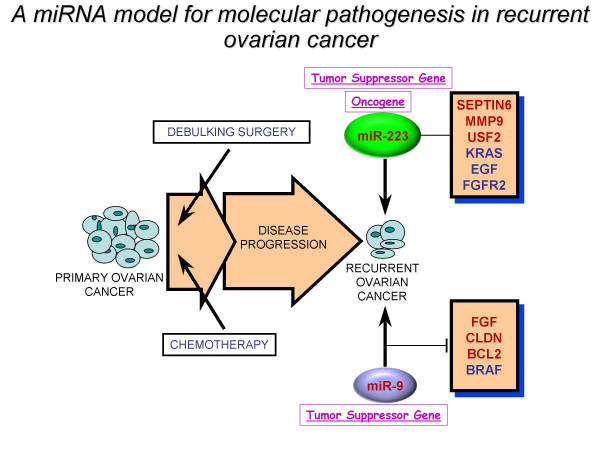
A miRNA model for the molecular pathogenesis of recurrent ovarian cancer. Based on integrated transcriptome and miRNA analysis, we identified a "recurrent metastatic signature" comprised top dysregulated miRNAs, miR-9 (down) and miR-223 (up) that correlate with our previous transcriptome findings. miR-9 appears to be a tumour suppressor gene and when downregulated, it can lead to drug resistance. miR-223 can act either as oncogene or tumour suppressor gene. Genes highlighted in red were previously identified in our transcriptome study. Genes in blue have been well characterised in ovarian cancer. FGF signalling pathway appears to be commonly shared between our identified miRNAs.

In summary, we propose a miRNA model for the molecular pathogenesis of recurrent ovarian cancer (Figure 9). Some of the differentially deregulated miRNAs identified correlate with our previous transcriptome findings. Based on integrated transcriptome and miRNA analysis, miR-9 and miR-223 can be of potential importance as biomarkers in recurrent ovarian cancer, given their abundance, stability and easy detection. Future use of this miRNA signature as a blood-based diagnostic and prognostic tool can ultimately be translated into designing drugs that might offer a promising treatment to selected ovarian cancer patients.

## Competing interests

The authors declare that they have no competing interests.

## Authors' contributions

AL performed the experiments and drafted the manuscript. SOT performed extensive editing and preparation of manuscript. MR assisted with cutting frozen sections. RF and CB examined the pathological specimens. RF also assisted with LCM and data analysis. LK contributed to RT-PCR analysis of Dicer and Drosha. NG, TDA and EMG provided the tumour samples. SF and ML aided in IHC analysis. CM, OS and BS assisted with critical examination of the manuscript. JOL supervised experimental work and manuscript editing. Final manuscript was read and approved by all authors.

## Supplementary Material

Additional file 1 miRNA genes differentially expressed between recurrent and primary serous papillary ovarian adenocarcinomas in the training cohort with their numerical fold changes ranging from 2 to 11. Common miRNA genes highlighted in red have been identified as differentially expressed in diverse cancers. This signifies that specific miRNAs are involved in common molecular pathways. miRNAs highlighted in green were the top dysregulated in recurrent vs primary serous papillary adenocarcinomas in the training cohort. Click here for file
